# About Gray Hair

**Published:** 1885-04

**Authors:** 


### About Gray Hair.

The heroine of a popular play is made to exclaim that she never considers a man old until he is fat and bald.

She was quite right in omitting gray hair as a sign of age. Though it is generally esteemed so, there are too many exceptions on both sides to allow it as an infallible sign.

Many persons begin to show gray hairs while they are yet in their twenties, and some while in their teens. This does not by any means argue a premature decay of the constitution. It is a purely local phenomenon, and may co-exist with unusual bodily vigor. The celebrated author and traveler George Borrow turned quite gray before he was thirty, but was an extraordinary swimmer and athlete at sixty-five.

Many feeble persons, and others who have suffered extremely, both mentally and physically, do not blanch a hair until past middle life; while others, without assignable cause, lose their capillary coloring matter rapidly when about forty years of age.

Race has a marked influence. The traveler, Dr. Orbigny, says that in the many years he spent in South America he never saw a bald Indian, and scarcely ever a gray-haired one. The negroes turn more slowly than the whites. Yet we know a negress of pure blood, about thirty-five years old, who is quite gray.

In this country, sex appears to make little difference. Men and women grow gray about the same period of life.

In men the hair and beard rarely change equally. The one is usually darker than the other for several years; but there seems no general rule as to which whitens first.

The spot where grayness begins differs with the individual. The philosopher, Shopenhauer, began to turn gray on the temples, and complacently framed a theory that this is an indication of vigorous mental activity.

The correlation of gray hair, as well as its causes, deserves more attentive study than they have received. Such a change is undoubtedly indicative of some deep-seated physiological process, but what this is we can only ascertain by a much wider series of observations than have yet been submitted to scientific analysis.

